# Supplementary figures and images for: Visualization of translocons in Yersinia type III protein secretion machines during host cell infection
Source: PLoS Pathog. 2018 Dec 26;14(12):e1007527. doi: 10.1371/journal.ppat.1007527 (PMC6324820; doi:10.1371/journal.ppat.1007527)

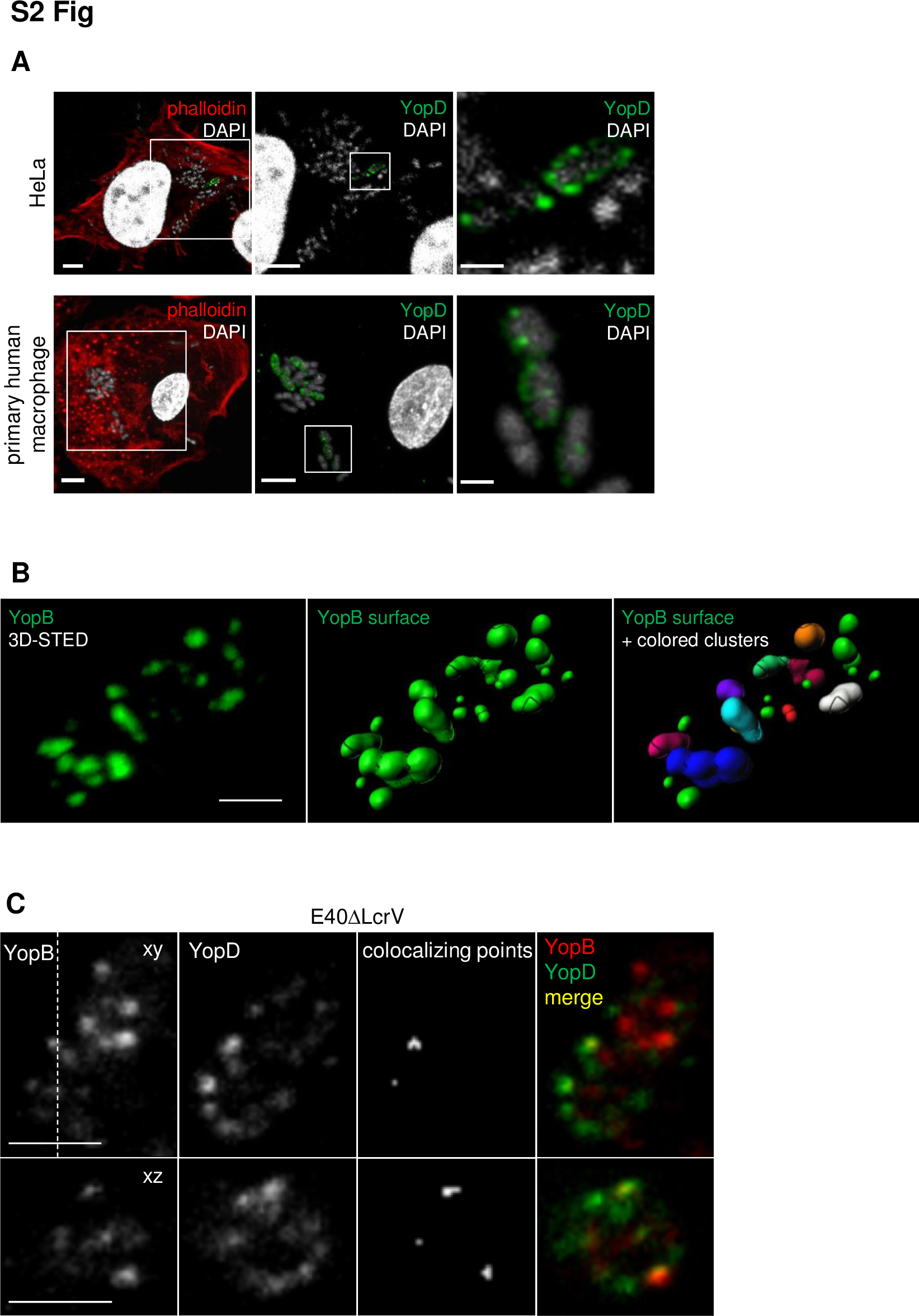

Supplement: S2 Fig — (A) HeLa cells (upper row) were infected with WA-314 at a MOI of 100 for 2 h. Primary human macrophages (lower row) were infected with WA-314 at a MOI of 10 for 20 min. Cells were stained with anti-YopD antibody, phalloidin and DAPI. Representative confocal images are depicted. Boxed regions depict positions of the enlargements in images to the right. Scale bars (from left to right): 5 μm, 5 μm and 1 μm. (B) YopB spots concentrate in clusters. myc-Rac1Q61L transfected HeLa cells were infected with WA-314 at a MOI of 50 for 60 min and stained with anti-YopB antibody and Abberior635P secondary antibody. Z-stacks were recorded in 3D-STED mode and YopB spots on individual bacteria were subjected to image analysis (Methods). A representative 3D-STED recording is depicted as original fluorescence staining (green, left) and as segmented surface representation (green, middle). Surface representations were used for 3D analysis of YopB spots in Imaris. Clusters formed by at least 2 spots were coded in different colors and the residual spots remained green. Scale bar: 1 μm (C) STED imaging of YopB and YopD during Yersinia E40ΔLcrV infection. E40ΔLcrV infected HeLa cells were co-immunostained for YopB (secondary antibody AlexaFluor-594) and YopD (AbberiorStarRed). All images show representative single planes of z-stacks recorded in 3D-STED mode. xz projection at the level of the dashed line. Representation of colocalizing points was generated using the “Colocalization” plugin in ImageJ. Merge (yellow) of green (YopD) and red (YopB) fluorescence. Scale bar: 1 μm. (TIF) [file ppat.1007527.s002.tif]

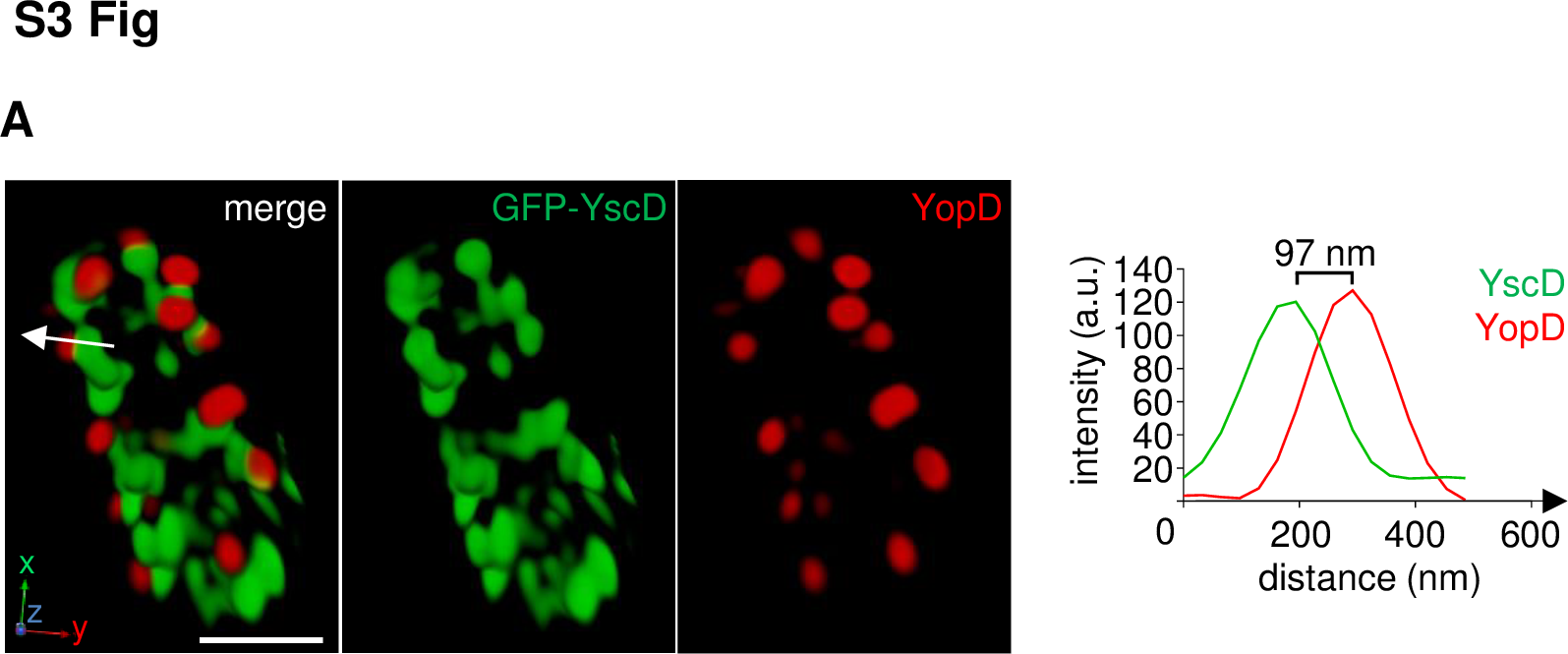

Supplement: S3 Fig — HeLa cells were infected with Y. enterocolitica E40 GFP-YscD, stained with anti-YopD antibody and z-stacks of YopD positive bacteria were recorded with SIM. A 3D reconstruction of a representative bacterium is depicted. Scale bar: 1 μm. Fluorescence intensity profiles along the longitudinal axis (arrow) of a GFP-YscD/YopD pair indicate a distance of 97 nm between fluorescence maxima. (TIF) [file ppat.1007527.s003.tif]

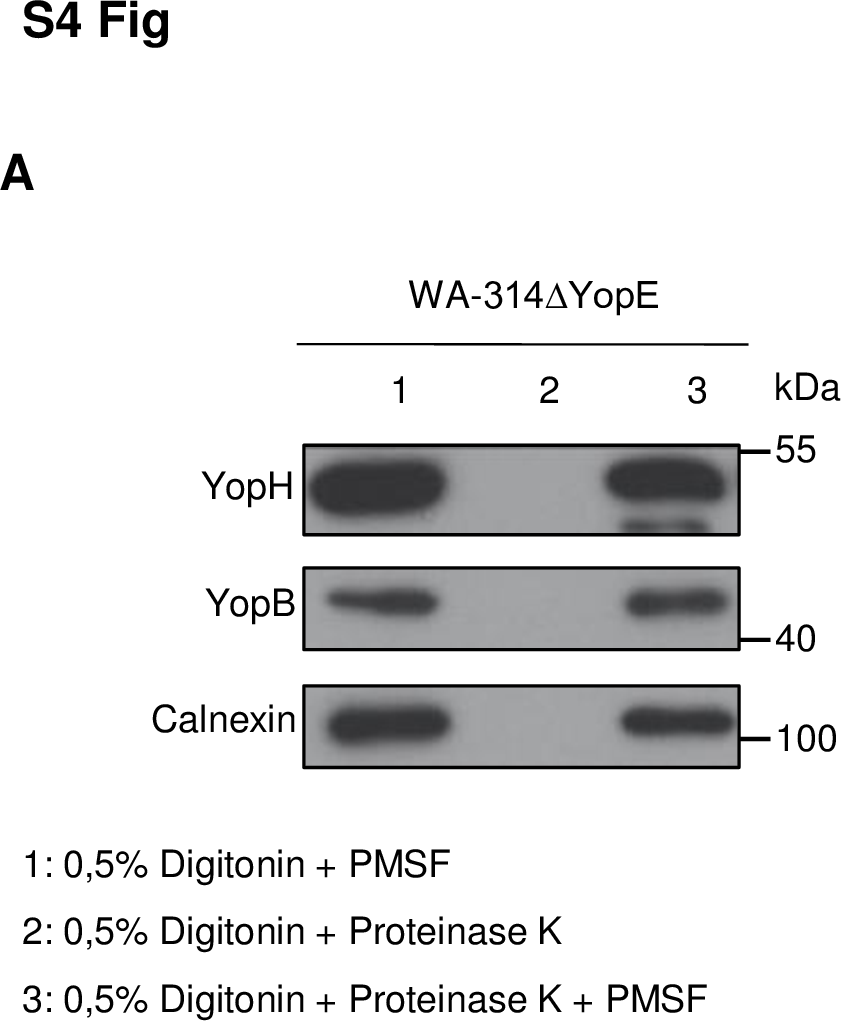

Supplement: S4 Fig — HeLa cells were infected with WA-314ΔYopE at a MOI of 100 for 60 min. To demonstrate the capability of PK to degrade Yops and of PMSF to efficiently inhibit PK, digitonin plus PMSF, digitonin plus PK or digitonin plus premixed PK+PMSF were added to the infected cells before centrifugation and immunoblotting of the supernatant for YopH, YopB and calnexin. (TIF) [file ppat.1007527.s004.tif]

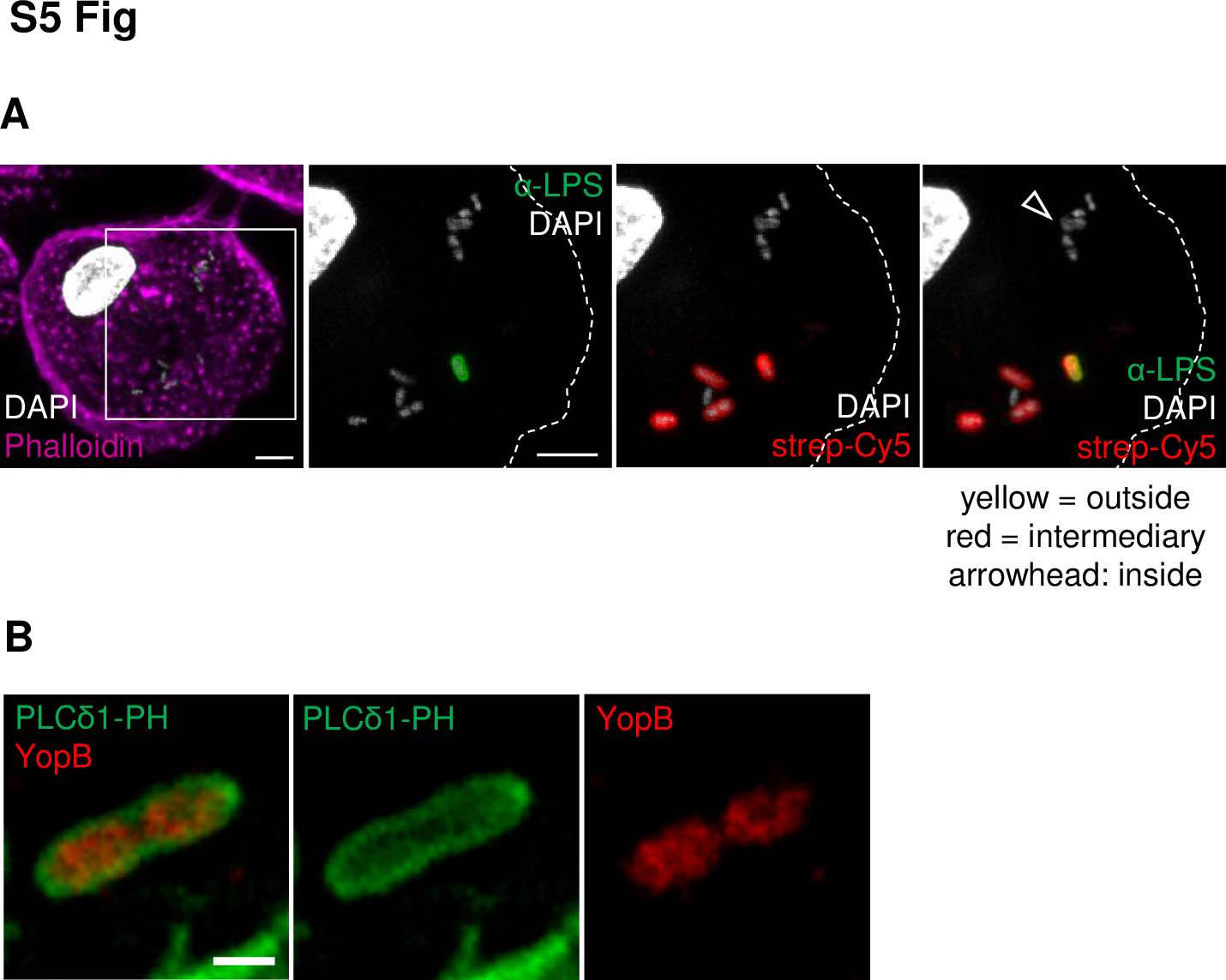

Supplement: S5 Fig — (A) Yersinia in different stages of internalization during infection of primary human macrophages. Macrophages were infected with surface-biotinylated WA-314 at a MOI of 10 for 20 min and stained with anti-LPS antibody and streptavidin-Cy5 without cell permeabilization. Then cells were permeabilized and stained with fluorescent phalloidin and DAPI. From left to right: 1. Overview of infected macrophage. 2.–4. Enlargement of bacteria located outside (yellow in merge), in the intermediary compartment (red in merge) and in the inside compartment (white in merge, arrowhead). Scale bars: 5 μm. (B) PLCδ-PH-GFP enrichment around YopB positive bacteria in human macrophages. Primary human macrophages expressing PLCδ-PH-GFP were infected with WA-314 at a MOI of 10 for 20 min and immunostained for YopB. Scale bar: 1 μm. (TIF) [file ppat.1007527.s005.tif]

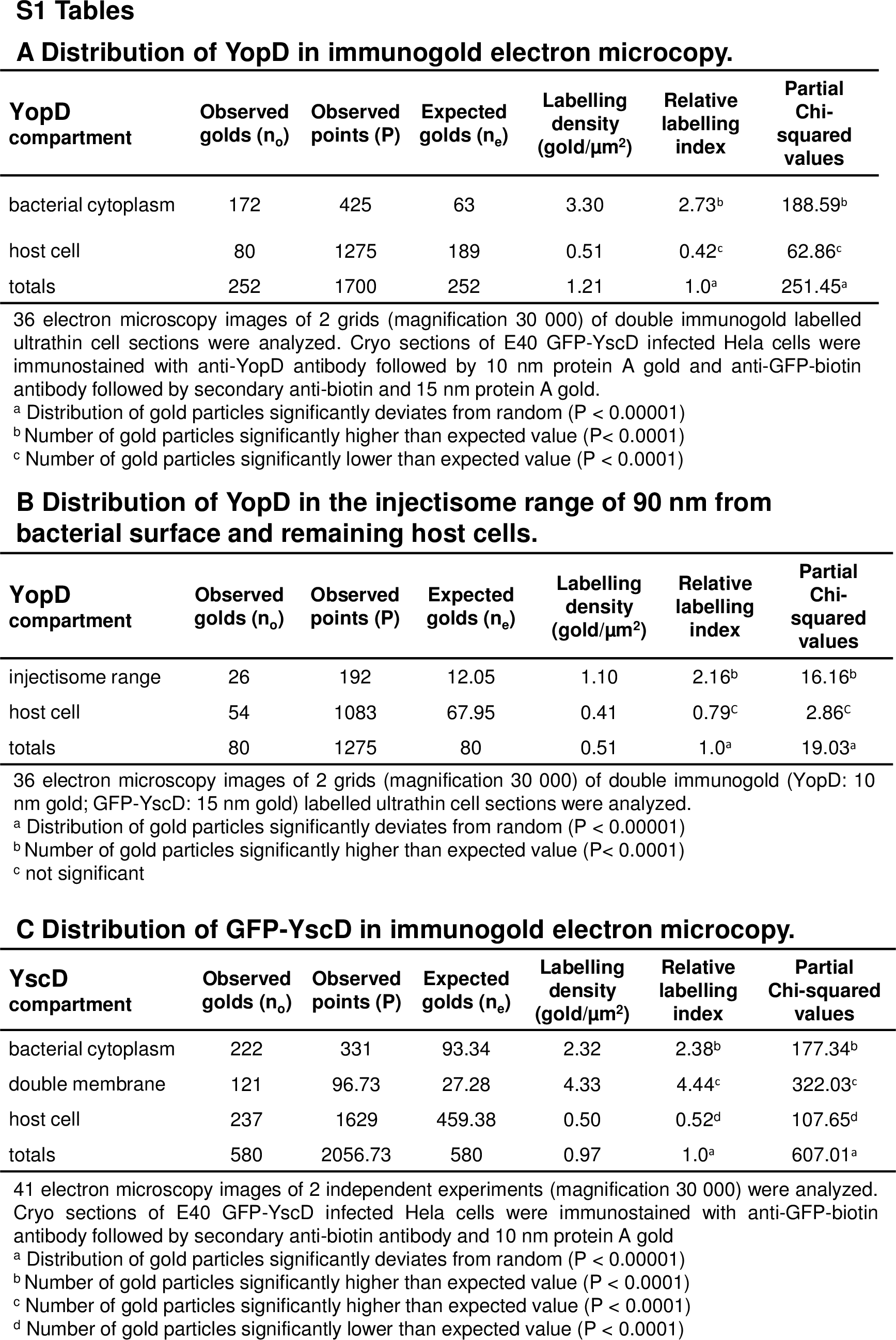

Supplement: S1 Table — (A) Distribution of YopD in immunogold TEM of Yersinia infected cells. 36 electron microscopy images of 2 grids (magnification 30.000) of double immunogold labelled ultrathin cell sections were analyzed. Cryo sections of E40 GFP-YscD infected HeLa cells were immunostained with anti-YopD antibody followed by 10 nm protein A gold and anti-GFP-biotin followed by secondary anti-biotin and 15 nm protein A gold. a Distribution of gold particles significantly deviates from random (P < 0.00001) b Number of gold particles significantly higher than expected value (P< 0.0001) c Number of gold particles significantly lower than expected value (P < 0.0001). (B) Distribution of YopD between the injectisome range of 90 nm from bacterial surface and adjacent host cells. 36 electron microscopy images of 2 grids (magnification 30.000) of double immunogold (YopD: 10 nm gold; GFP-YscD: 15 nm gold) labelled ultrathin cell sections were analyzed. a Distribution of gold particles significantly deviates from random (P < 0.00001) b Number of gold particles significantly higher than expected value (P< 0.0001) c not significant. (C) Distribution of GFP-YscD in immunogold TEM. 41 electron microscopy images of 2 independent experiments (magnification 30.000) were analyzed. Cryo sections of E40 GFP-YscD infected HeLa cells were immunostained with anti-GFP-biotin followed by secondary anti-biotin and 10 nm protein A gold. a Distribution of gold particles significantly deviates from random (P < 0.00001) b Number of gold particles significantly higher than expected value (P < 0.0001) c Number of gold particles significantly higher than expected value (P < 0.0001) d Number of gold particles significantly lower than expected value (P < 0.0001). (TIF) [file ppat.1007527.s006.tif]
